# What is the extent of research on the characteristics, behaviors, and impacts of health information technology champions? A scoping review

**DOI:** 10.1186/s12911-016-0240-4

**Published:** 2016-01-12

**Authors:** Christopher Michael Shea, Charles M. Belden

**Affiliations:** CB# 7411 McGavran-Greenberg Hall, Gillings School of Global Public Health, University of North Carolina-Chapel Hill, Chapel Hill, NC 27599-7411 USA

**Keywords:** Health information technology, Champion, Implementation science, Organizational innovation, Organizational change

## Abstract

**Background:**

Although champions are commonly employed in health information technology (HIT) implementations, the state of empirical literature on HIT champions’ is unclear. The purpose of our review was to synthesize quantitative and qualitative studies to identify the extent of research on the characteristics, behaviors, and impacts of HIT champions. Ultimately, our goal was to identify gaps in the literature and inform implementation science.

**Methods:**

Our review employed a broad search strategy using multiple databases—Embase, Pubmed, Cinahl, PsychInfo, Web of Science, and the Cochrane library. We identified 1728 candidate articles, of which 42 were retained for full-text review.

**Results:**

Of the 42 studies included, fourteen studies employed a multiple-case study design (33 %), 12 additional articles employed a single-case study design (29 %), five used quantitative methods (12 %), two used mixed-methods (5 %), and one used a Delphi methodology (2 %). Our review revealed multiple categories and characteristics of champions as well as influence tactics they used to promote an HIT project. Furthermore, studies have assessed three general types of HIT champion impacts: (1) impacts on the implementation process of a specific HIT; (2) impacts on usage behavior or overall success of a specific HIT; and (3) impacts on general organizational-level innovativeness. However the extent to which HIT projects fail even with a champion and why such failures occur is not clear. Also unclear is whether all organizations require a champion for successful HIT project implementation. In other words, we currently do not know enough about the conditions under which (1) a health IT champion is needed, (2) multiple champions are needed, and (3) an appointed champion—as opposed to an emergent champion—can be successful.

**Conclusions:**

Although champions appear to have contributed to successful implementation of HIT projects, simply measuring the presence or absence of a champion is not sufficient for assessing impacts. Future research should aim for answers to questions about who champions should be, when they should be engaged, what they should do, how management can support their efforts, and what their impact is given the organizational context.

## Background

The promise of increased health information access and information exchange for improving health systems and patient outcomes has prompted efforts within many countries to enhance the health information technology (HIT) infrastructure. For example, in the United States, financial incentives through the Medicare and Medicaid programs for “Meaningful Use” of electronic health records (EHR) are contributing to substantial increases in adoption of EHRs and related HIT. Between 2009 and 2012, physician adoption of EHR technology used to demonstrate five Meaningful Use core objectives increased by approximately 66 % [1], and by the end of 2012, more than 75 % of all eligible U.S. hospitals had qualified for financial incentives [2]. As of July 2015, eligible hospitals and providers had received more than $31B in Meaningful Use incentive payments [3]. In addition to Meaningful Use, value-based payment and care delivery models in the U.S., such as Accountable Care Organizations, are increasing the need for health care providers and organizations to exchange patient information electronically [4], thus incentivizing the adoption of interoperable EHR systems.

Although adoption of EHRs and other HIT is increasing, implementation of these innovations carries substantial risk due to the financial investment required, the potential to negatively affect the provider and patient experience, and the opportunity cost of failure. Furthermore, implementing new health information systems is complex because these systems affect multiple organizational members and work processes [5]. Because of the risk and complexity involved, organizations are typically advised to undergo a careful planning process and dedicate adequate financial and human resources in order to deploy successful implementation strategies [6, 7].

Numerous approaches for implementing HIT and similar innovations in health care organizations have been documented, but there is mixed evidence about which are most important for promoting consistent and appropriate use of these innovations [7, 8]. Having a champion to promote an innovation and support the requisite change effort (i.e., “fight for the cause”) is one approach commonly cited in the literature and used in practice [9, 10]. Despite being commonly employed in HIT implementations, however, champions may not be well understood. For example, several years ago Howell and Higgins, whose work was not health specific, identified that research has not sufficiently examined the characteristics of champions or how their roles may (or may not) differ from other organizational members in the implementation process [11, 12]. Now, approximately 25 years after Howell and Higgins made these observations, the current state of the literature on HIT champions, in particular, is unclear. This lack of clarity impedes development of evidence-based approaches for identifying champions, supporting the efforts of champions, and assessing the impacts of champions. The purpose of our review was to synthesize empirical studies, both quantitative and qualitative, to identify the extent of research on the characteristics, behaviors, and impacts of HIT champions. Ultimately, our goal was to identify gaps in the literature on HIT champions to inform future research.

## Methods

We conducted a scoping review, which “provides a preliminary assessment of the size and scope of available research literature” [13]. Scoping reviews are similar to systematic reviews with respect to search methodology and approach to describing a body of literature. However, systematic reviews typically have more specific research questions than do scoping reviews. Also, systematic reviews use methodologies to assess the quality of articles included in the review, whereas scoping reviews typically do not [14]. We chose to conduct a scoping review because we anticipated that HIT champions had not been well studied and that there would not be a sufficient evidence base to answer specific research questions about HIT champion impacts. The methods we employed were similar to those recommended for scoping studies by Levac et al., for example (1) combining a broad research question with a clearly articulated scope and clear definitions of key concepts; (2) using an iterative, team-oriented approach to study selection and data abstraction; and (3) drawing upon qualitative analytical techniques to identify themes in the literature [15].

### Data sources and searches

We conducted searches for relevant studies in Embase, Pubmed, Cinahl, PsychInfo, Web of Science, and the Cochrane library through September 2014. Prior to submitting the manuscript for review, we conducted an additional search to determine whether additional relevant articles had been published since our original search. For our review, we defined champion as an individual who is a member of an organization (i.e., internal) and facilitates the change necessary to implement a new HIT system within the organization. This definition is consistent with Schon’s seminal definition of product champion [16]. Although champion is distinct from concepts such as change agent and opinion leader [17], we anticipated that some authors might use such terms synonymously. Therefore, we used a broad range of search terms: champion, change agent, innovator, opinion leader, super user, entrepreneur, leader, and boundary spanner. In addition to our database searches, we manually forward-searched references of articles that met our inclusion criteria to identify additional articles that might be relevant but did not appear in our database search results.

### Study selection

Because we were particularly interested in champions of HIT implementation, we included only articles about such champions, rather than using the broader strategy of including champions of any type of organizational change (e.g., an educational intervention) or champions of IT in all types of settings (e.g., manufacturing companies). We did so because implementing IT, unlike some other types of innovations, requires learning to use a new technology and modifying existing work processes; therefore, IT implementation may require different champion characteristics and tactics as compared to other innovations. Also, health care organizations are organized and staffed in ways that are substantially different from most other types of organizations and provide services to clients (patients) that are not analogous to other types of products/services. In summary, studies of changes unrelated to IT in healthcare settings, or of IT implementations in non-health settings, likely would yield some results not applicable to champions of HIT.

Studies were excluded from full-text review if they did not meet the following criteria:

1. Written in English
2. Published in 1990 or later
3. Abstract available
4. Empirical study using qualitative or quantitative methods
5. Investigates development, implementation, and/or use of health information technology. HITECH Act definition of health information technology: “hardware, software, integrated technologies, or related licenses, intellectual property, upgrades, or packaged solutions sold as services that are designed for or support the use by health care entities or patients for the electronic creation, maintenance, access, or exchange of health information” [18]. Examples include technologies involving clinical notes, medication lists, radiology and laboratory results, alerts, and telemedicine.
6. Includes analysis of a champion that is internal to the organization implementing the HIT.

**Fig. 1:**
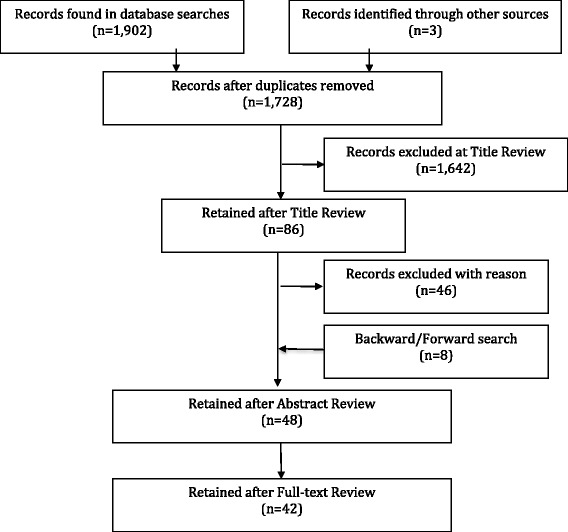
PRISMA diagram for the HIT champion literature search

This study used the PRISMA statement flow chart to analyze literature search results (Fig. 1). After one author (CB) reviewed abstracts using the inclusion criterion 1 through 4 (listed above), a 20 % random sample of the abstracts was selected and reviewed by the other author (CS) to validate the inclusion/exclusion process. Using inclusion criteria 1–6, both authors (CB and CS) then completed a full-text review of articles that were not excluded during abstract review.

### Data extraction

Articles selected for full-text review were charted [15, 19] using a charting form developed in an Excel spreadsheet [19, 20]. We adapted the Howell & Higgins framework [11] to determine which variables were needed to answer the research questions. We then iteratively developed the charting form used to extract the variables from each study. One member of the research team (CB) extracted data from all articles included in the review, and the other team member (CS) performed the same process on a sample of articles [15]. The charting form included the following fields for each study:

- Author, year, setting, study design, sample
- Type of HIT studied
- Whether the term “champion” was used. If so, whether “champion” was explicitly defined; if not, which term was used instead of champion.
- Champion characteristics (e.g., role in the organization)
- Champion influence tactics (e.g., building coalitions)
- Management support for the champion (e.g., supporting pilot projects)
- Champion impact (e.g., on adoption)

Once the charting process was complete, we synthesized the results to develop summary findings pertinent to the variables in the charting form. Consistent with the stated purpose of our review, we then considered these summary findings in the context of current HIT implementation practice in order to develop recommendations for future research [15].

## Results

### Study selection

Our database search identified 1902 articles, and we identified three additional articles from forward searches on Howell and Higgins [11]. After removing duplicates, we were left with 1728 candidate articles. We were unable to locate three of these papers [21–23]. After title review, we excluded 1642 of the articles. Agreement on exclusion of studies at this stage was 99 % in the 20 % random sample (*n* = 344) reviewed by both authors. After abstract review, 48 articles remained for full-text review, during which 6 additional articles were excluded.

### Study characteristics

Table 1 summarizes the settings, methods, and main findings of the articles included in our review. Of the 42 articles included in our full-text review, 34 (81 %) used the term champion, and 8 (19 %) provided an explicit definition of the term champion. Articles that did not use the term champion used terms such as change agent [24], bilingual coach [25], opinion leader [26, 27], innovator [28], facilitator [29], and super user [30]. Despite the variation in terms used, we were able to ascertain that each of the 42 studies included in our review described an HIT champion as we defined the concept, i.e., an individual who is a member of an organization (i.e., internal) implementing a new HIT system within the organization. Fourteen studies employed a multiple-case study design (33 %), 12 additional articles employed a single-case study design (29 %), five used quantitative methods (12 %), two used mixed-methods (5 %), and one used a Delphi methodology (2 %). Below we synthesize the characteristics, behaviors, and impacts of IT champions from studies included in our review. Table 2 summarizes this information for each study.

**Table 1:** Summary of articles included in the review of HIT champions (*n* = 42)

Reference	Intervention	Setting	Evaluative method	Objectives	Summary findings
Al-Qirim [32]	Telehealth	One Dermatology Department in New Zealand.	Case study	Explain factors influencing the adoption and diffusion of telemedicine for dermatology.	Study emphasized the importance of having a product champion for the adoption of a telemedicine initiative in New Zealand.
Al-Qirim [33]	Telehealth	One Psychiatry Department, and one Dermatology Department in New Zealand.	Case study	Explain factors influencing the adoption and diffusion of telemedicine for psychiatry and dermatology.	Study emphasized the importance of having a product champion for the adoption of a telemedicine initiative in New Zealand.
Andre, Ringdal, Loge, Rannestad, Kaasa [63]	Handheld symptom assessment	Oncology Department in University Hospital (Norway)	Case study	Examine responses and attitudes of users, and factors promoting implementation.	Lack of knowledge about the formal project aims, training and communication with organizational leaders were barriers to implementation.
Ash, Stavri, Dykstra, Fournier [31]	Computerized physician order entry (CPOE)	One Teaching hospital in Virginia (USA), Veteran’s health care system in Washington (USA), one non-profit hospital in California (USA)	Multiple case study	Identify factors associated with the implementation of CPOE in inpatient and outpatient settings.	Three types of “special people” have been identified as key personnel in the implementation of CPOE.
Ash, Gorman, Lavelle, Payne, Massaro, Frnatz, Lyman [34]	Computerized physician order entry (CPOE)	University of Virginia (USA), Veterans Affairs Puget Sound Health Care System (USA), El Camino Hospital (USA)	Observation, interviews, and focus groups	Describe perceptions of CPOE among diverse professionals in sites with successful CPOE implementation	Four themes: organizational issues (e.g., collaboration); clinical/professional issues; technical/IT implementation issues; organization of information
Ash, Sittig, Wright [57]	Clinical decision support (CDS)	Independent physician organization in Oregon (USA).	Case study	Identify barriers and facilitators of CDS implementation in a community setting.	Joint selection and purchase, and implementation of EHRs with CDS; centrally managed EHR, and improved data are necessary.
Ash, Sittig, Guappone, Dykstra, Richardson, Wright, Carpenter, McMullen, Shapiro, Bunce, Middleton [47]	Clinical decision support (CDS)	Two community hospitals and five ambulatory clinics in the US.	Multiple case study	Identify factors associated with implementation of CDS.	Workflow integration, well designed user interfaces, knowledge management, and intentional interaction among stakeholders are key factors in CDS implementation.
Carlfjord, Lindberg, Andersson [35]	Computer-based lifestyle intervention.	Six primary health care (PHC) centres in Sweden.	Multiple case study	Explore organizational members’ perceptions and usefulness of a computer-based lifestyle intervention.	Extra resources, such as manpower, and committed leadership are key factors in implementation.
Chedid, Golden, Jager [56]	University of Chicago Medicine’s Diabetic Retinopathy Screening Program	Chicago Family Health Center (Federally qualified health center).	Case study	Identify operational challenges in the implementation of a diabetic retinopathy screening program.	Strong physician leadership is a key element for the implementation of an HIT screening intervention.
Cresswell, Sadler, Rodgers, Avery, Cantrill, Murray, Sheikh [24]	Pharmacist-led information technology intervention.	34 primary care organizations in central England (UK).	Case Study	Understand the organizational and social environment of a pharmacist-led information technology intervention.	Face-to-face contact with practice staff, and a designated champion were keys to implementation of pharmacy HIT.
Crosson, Etz, Wu, Straus, Eisenman, Bell [42]	Electronic prescribing	Five primary care practices in the US.	Multiple Case Study	Identify the factors associated with implementation of electronic prescribing.	Implementation of electronic prescribing requires workflow redesign, and improved health information exchange.
Culler, Jose, Kohler, Edwards, Dee, Sainfort, Rask [45]	Inpatient pharmacy system.	Two pediatric hospitals – Egleston Children’s Health Care and Scottish Rite Children’s Medical Center (UK).	Case study	Describe the facilitators and barriers to the implementation of an inpatient pharmacy system.	Training super-users, extensive pre-implementation training, formal feedback mechanisms, and technical support following deployment are facilitators of HIT.
Feldman, Schooley, Bhavsar [49]	Health Information Exchange (HIE)	Health system in Virginia	Case study	Investigate technical, organizational, and governance of HIE implementation	Project champions play a key decision- making role in governance
Feldstein, Schneider, Unitan, Perin, Smith, Nichols, Lee [58]	Decision support system – Patient Panel Support Tools (PST)	Non-profit group model HMO, Kaiser Permanente Northwest, in Washington and Oregon (US).	Case study	Examine health care workers attitudes toward the adoption and use of a Patient Panel Support tool.	Implementation required roles for non-PCP staff, leadership, training, and dedicated time for using the HIT.
Gagnon, Desmartis, Labrecque, Legare, Lamothe, Fortin, Rancourt, Duplantie [36]	Electronic Medical Record (EMR)	Family medicine group (FMG) in Quebec, Canada.	Case study	Explore factors that influence the implementation of an EMR.	Organizational factors such as presence of a champion, innovative culture, personal characteristics, and a scientifically based implementation strategy are important.
Garfield and Watson [50]	Telehealth	State telemedicine initiatives in Georgia, Pennsylvania, Ohio, and Wisconsin.	Multiple case study	Examine factors contributing to the success of telemedicine initiatives.	‘Technical’ and ‘user champions’ may be necessary to implement telemedicine interventions.
Gordon, Camhi, Hesse, Odlum, Schnall, Rodriguez, Valdez, Bakken [25]	Continuity of Care Document (CCD)/Personal Health Record (PHR).	NYPS SelectHealth HIV/AIDS care sites in New York City (USA).	Case study	Examine the processes and outcomes of a Continuity of Care Document/PHR for people living with HIV/AIDS.	Training and organizational commitment are important factors in implementation of a PHR.
Greiver, Barnsley, Glazier, Moineddin, Harvey [37]	Electronic Medical Records	18 community-based family physician practices in Toronto, Canada.	Case study	Examine factors influencing the implementation of electronic medical records.	EMR implementation was also influenced by lack of leadership, relative advantage, high complexity, low compatibility, and available organizational slack.
Halbesleben, Wakefield, Ward, Brokel, Crandall [51]	Clinical Information System (CIS). Includes an electronic health record (EHR) with computerized physician order entry (CPOE).	Large, Midwestern rural referral hospital.	Case study	Explore the impact of Super Users on the implementation of a CIS.	Super-users, and leadership support for super users are important factors in implementation of (CIS).
Hao, Padman, Telang [26]	Mobile Clinical Access Portal (MCAP) with secured wireless PDA-based solution providing access to electronic medical record system (EMR).	Community-based healthcare system in southwestern Pennsylvania.	Multivariate regression analysis	Examine empirical evidence for the contextual factors associated with physician adoption of a PDA-based electronic medical record system.	Opinion leaders have significant effects on physician adoption of PDA-based EHR systems.
Hartswood, Procter, Rouchy, Rouncefiled, Slack, Voss [29]	Improved electronic tools for management of patient records and patient care.	Toxicology ward of a large hospital.	Case study	Explore the process of participant design of health information technology.	HIT professionals must design and develop systems with users.
Hendy and Barlow [43]	Telehealth	Three health and social care organizations with experience in telehealth in England (UK).	Multiple case study	Examine the role of champions in telehealth initiatives.	Questions the positive and necessary role that champions play in HIT implementation.
Hsiao, Li, Chen, Ko [23]	Mobile nursing information system	Eighty-four Nursing Directors at Hospitals in Taiwan	Multivariate regression analysis	Examine the factors associated with adoption of MNIS	Organizations should scan environment, identify mobile nursing needs, and develop vendor relationships in order to adopt.
Leidner, Preston, Chen [28]	Hospital health information technology.	Matched pairs of CIOs and executives.	Multivariate regression analysis	Examine the hospital characteristics associated with IT innovation.	There are different types of CIO-Board dynamics that affect the role of CIO as a champion and his/her champion behaviors.
McAlearney, Schweikhart, Medow [48]	Handheld computers in clinical practice.	161 informants at seven clinical practice sites.	Multiple case study	Describe strategies that promote use of handheld computers.	Organizations will use different strategies that promote handheld computer use and remain responsive to physician needs.
Miller and Sim [46]	Electronic medical record (EMR).	EMR managers in physician champions in 30 physician organizations.	Multiple case study	Identify key barriers to physician’s use of EMRs.	Practices without physician champions are likely to struggle to achieve quality or financial benefits.
Novak, Anders, Gadd, Lorenzi [52]	Barcode medication administration (BCMA)	Multi-hospital, tertiary medical center in the US.	Case study	Examine mediators efforts to implement BCMA	Clinicians can improve the safety and effectiveness of BCMA implementation with institutional support.
Paré, Elam, Ward [38]	Patient charting system (PCS).	Burn center of a large, not-for-profit teaching hospital.	Case study	Examine the implementation of a patient charting system.	Patient charting system implementation requires key actors anticipating and proactive with challenges; key actors are associated with quality of implementation; PCS implementation is indeterminate process, and outcomes are associated with management of the process and actions after introduction of system.
Paré, Sicotte, Jaana, Girouard [41]	Clinical information system (CIS).	Participants with backgrounds in CIS project management.	Delphi study	Identifying the risks associated with the implementation of CIS.	Risk management is a key strategy throughout the implementation of CIS.
Paré, Sicotte, Poba-Nzaou, Balouzakis [61]	Mobile computing technology; Clinical information system (CIS).	Future users of mobile computing technology in home care organizations; and a large teaching hospital implementation of CIS.	Multivariate regression analysis	Examine clinicians’ early perceptions of organizational readiness for change with clinical information system projects.	Organizational readiness for change is a key factor in clinician’s initial support for implementation of CIS.
Piscotty, Tzeng [59]	Clinical information system (CIS)	Regional multi-hospital system.	Multiple case study	Explore CIS readiness activities adopted by chief nurse executives.	Chief nurse executives suggested that champions are necessary at multiple organizational levels to obtain buy in and gather support for implementation.
Poe, Abbott, Pronovost [55]	Electronic health records (EHR)	Clinical units at an academic medical center (*n* = 9).	Structured program evaluation	Evaluate the effectiveness of peer coaches impact on increasing learner satisfaction and confidence in EHR use.	Peer coaches had a positive effect on satisfaction and confidence.
Postema, Peeters, Friele [44]	Telehealth	Care organizations in the Netherlands (*n* = 3).	Multiple case study	Examine the key factors that improve implementation of video communication.	Technical stability and the alignment of the external environment with organizational goals and implementation strategy are key factors.
Shachak, Montgomery Dow, Barnsley, Tu, Jadad, Lemieux-Charles [74]	Electronic medical records (EMR)	Four family health teams, one family health organization (Canada).	Multiple case study	Investigate user expectations and needs for end-user support for EMR.	Highlights importance of on-site support and super-users in liaison roles, local development of support practices, and gaps in understanding of other organizational members’ work processes.
Sharkey, Hudak, Horn, Barrett, Spector, Limcangco [60]	Clinical decision support tools for pressure ulcer prevention.	Nursing homes in Washington, D.C. (USA) (*n* = 14).	Multivariate regression analysis	Examine nursing home factors associated with implementation of clinical decision support tools for pressure ulcer prevention.	High involvement of nurse managers, in-house dietitian, high participation from staff educator and QI personnel, internal champions, and openness to redesign were associated with implementation.
Shaw, Howard, West, Crabtree, Nease, Tutt, Nutting [39]	EPIC quality improvement intervention.	Primary care practices in Colorado (USA) (*n* = 14)	Multiple case study	What are the roles of champions in the implementation of organizational innovations.	Two types of champions are key – specific project champions, and organizational change champions.
Sloane, Wroth, Halladay, Bray, Spragens, Stearns, Zimmerman [40]	Quality monitoring and reporting initiative.	Primary care practices in North Carolina (USA)	Multiple case study	Examine the factors that impact initiation and maintenance of a quality monitoring and reporting process.	Complex sets of factors are required to implement and sustain quality-reporting interventions.
Verhoeven, Steehouder, Hendrix, van Gemert-Pijnen [27]	Website with infection control guidelines.	Health care workers at 5 occupational groups in 4 hospitals in the Netherlands and Germany (*n* = 20).	Multiple case study	Identify factors impacting health care workers use of a website for communicating infection control guidelines.	Organizational factors play the key role in implementation, and inclusion of the health care workers is essential in the design process.
Wright, Ash, Erickson, Wasserman, Bunce, Stanescu, St Hilaire, Panzenhagen, Gebhardt, McMullen, Sittig [53]	Clinical decision support (CDS)	Two community hospitals; an academic medical center and outpatient clinic; Veterans Administration hospital outpatient clinics; community outpatient independent physician association; and academic outpatient clinic.	Multiple case study	Examine activities in the implementation of CDS.	Implementation of CDS requires a variety of skills and activities.
Yuan, Bradley, Nembhard [30]	Electronic health records (EHR)	Two medical units of a large academic hospital	Mixed methods case study	Investigate behaviors of EHR super-users.	Super-users may support or hinder implementation.
Yusof [64]	Critical care information system	Intensive care unit of tertiary referral center (Malayasia)	Case study	Examine factors associated with adoption of a critical care information system	Champions may be important, but other organizational factors may promote or hinder implementation.
Zandieh, Yoon-Flannery, Kuperman, Langsam, Hyman, Kaushal [62]	Electronic health records (EHR)	Practice managers (*n* = 11) and medical directors (*n* = 12) from ambulatory care network of large teaching hospital in New York City (USA).	Case study	Examine the different approaches to EHR implementation between organizations from paper-based vs. legacy EHRs.	Physician information technology champions was a priority for organizations with paper-based records implementing EHRs, but not a priority for those moving from a legacy EHR to a more sophisticated EHR.

**Table 2:** Summary of champion characteristics, influence tactics, and impacts

Reference	Champion type	Role in organization	IT experience or training	Champion was formal or emergent	Personality of champion	Tactics used by champion	Management support	Impact of champion
Al-qirim [32]	Clinical	Dermatologist	NA	Emergent	Achievement, innovative	NA	NA	Positive
Al-qirim [32]	Hybrid (clinical/admin)	Head of Dermatology	NA	Emergent	NA	Building coalitions	NA	NA
Al-qirim [33]	Admin	General Manager	NA	Emergent	Achievement, innovative	Appeal to higher authority, rational arguments	NA	NA
Al-qirim [33]	Clinical	Dermatologist	NA	Emergent	Achievement, innovative	NA	NA	Positive
Al-qirim [33]	Hybrid (clinical/admin)	Head of Dermatology	NA	Emergent	NA	Building coalitions, rational arguments	NA	NA
Ash et al. [34]	Varies	High-level leaders, non-clinicians, clinicians	Varies	NA	Achievement, innovative, persistence, persuasive	Building coalitions, rational arguments	Key factor	Positive
Ash et al. [57]	Clinical	Clinical champion	NA	Formal	NA	NA	NA	NA
Ash et al. [47]	Clinical	Non- clinicians, clinicians	Varies	NA	Charismatic, Innovative	Building coalitions, rational arguments	Yes	Positive
Carlfjord et al. [35]	NA	NA	NA	NA	Achievement, innovative	NA	Key factor	NA
Chedid et al. [56]	Clinical	Physician	NA	NA	NA	NA	NA	NA
Cresswell et al. [24]	Hybrid (clinical/admin)	Pharmacists	NA	Formal	NA	Individualized consideration	Key factor	Positive
Cresswell et al. [24]	Admin	Practice managers	NA	NA	NA	Individualized consideration	NA	Positive
Crosson et al. [42]	Varied (clinical/admin)	Physician	Yes	Formal	Innovative, persuasive	Charisma	NA	Positive
Culler et al. [45]	Clinical	Pharmacists	Yes	Formal	Persistence	Rational arguments	NA	Positive
Feldman et al. [49]	Hybrid (admin/technical)	Executive Vice President, Chief Technology Officer	NA	Formal	NA	Assertive actions, rational arguments	Key factor	NA
Feldstein et al. [58]	Varies	Staff	Yes	Formal	NA	NA	Key factor	NA
Gagnon et al. [36]	Hybrid (clinical/technical)	Physician	Yes	Formal	Achievement, innovative, persistence, persuasive	Assertive actions, building coalitions, Individual consideration, Ingratiation	NA	Positive
Garfield and Watson [50]	Technical	Telemedicine Coordinator	Yes	Formal	NA	NA	NA	Positive
Garfield and Watson [50]	Admin	President of MCG	NA	NA	NA	NA	NA	Positive
Greiver et al. [37]	NA	NA	NA	NA	Achievement, persuasive	NA	No	Positive
Halbesleben et al. [51]	Hybrid (clinical/admin)	Nurses, Nurse Managers	Yes	Formal	NA	NA	NA	Positive
Hao et al. [26]	Clinical	Physician	Yes	Informal	NA	NA	NA	Positive
Hartswood et al. [29]	Tech	IT staff	Yes	Formal	Persistence, persuasive	Bargaining	NA	NA
Hendy and Barlow [43]	NA	Telehealth Project Manager	Yes	NA	Achievement, innovative, persistence, persuasive	Building coalitions, intellectual stimulation, clandestine actions	NA	Varied
Hendy and Barlow [43]	NA	Project Manager	Yes	NA	Persistence, persuasive	Bargaining	NA	Varied
Leidner et al. [28]	Hybrid	CIO	Yes	Formal	Achievement, innovative	Assertive actions, Building coalitions	Varied	Varied
Leidner et al. [28]	Hybrid	CIO	Yes	Formal	Achievement, innovative	Rational arguments, bargaining	Varied	Varied
Leidner et al. [28]	Hybrid	CIO	Yes	Formal	Innovative	Rational arguments, bargaining	Varied	Varied
Leidner et al. [28]	Hybrid	CIO	Yes	Formal	Innovative	Assertive actions	Varied	Varied
McAlearney et al. [48]	Clinical	Physician	Yes	NA	Innovative	Building coalitions	NA	Positive
Miller and Sim [46]	Hybrid (clinical/technical)	Physician	Yes	NA	Innovative, persistent	Assertive actions	NA	Positive
Novak et al. [52]	Admin	Nursing Executive	NA	Informal	NA	Applying sanctions, friendliness and ingratiation	Yes	NA
Paré et al. [38]	Hybrid (clin/admin)	Nurse Managers	NA	Informal	Achievement, innovative, persistence, persuasive	Rational arguments	NA	Positive
Paré et al. [38]	Hybrid (clin/admin)	Medical Director	NA	Informal	Achievement, innovative, risk-taking, persuasive, persistence	Building coalitions, higher authority, sanctions, bargaining, assertive	NA	Positive
Paré et al. [41]	Clinical	Physician - medical director	NA	NA	Persistence, persuasive	NA	Key factor	NA
Paré et al. [61]		Nurses and Physicians	NA	NA	NA	NA	Yes	Varied
Piscotty and Tzeng [59]	Clinical	Nurse	Varies	NA	NA	Building coalitions	NA	NA
Poe et al. [55]	Clinical	Nurse - peer coach	Yes	Formal	NA	Ingratiation	NA	Positive
Postema et al. [44]	Hybrid (clinical/admin)	“Enthusiastic ambassadors”	NA	NA	Persistence, persuasive	Individualized consideration, rational arguments	Yes	Varied
Sharkey et al. [60]	Varied	Nurse/Mids/Admin	NA	NA	NA	NA	Yes	Positive
Shaw et al. [39]	Hybrid (clinical/admin)	Lead Physician	NA	Informal	Achievement, innovative	Assertive actions	NA	Positive
Shaw et al. [39]	NA	Team members	NA	Informal	NA	NA	NA	Positive
Shaw et al. [39]	Clinical	Physician Assistant	NA	Informal	NA	NA	Yes	Implementation failed
Shaw et al. [39]	NA	Various	NA	NA	NA	NA	NA	Implementation failed
Shaw et al. [39]	Hybrid (clinical/admin)	Med Director	NA	Formal	NA	NA	NA	Implementation failed
Shaw et al. [39]	Admin	Office Manager	NA	Formal	NA	NA	NA	Implementation failed
Shaw et al. [39]	Hybrid (clinical/admin)	Med Director	NA	Informal	Achievement, innovative	Building coalitions	Yes	Positive
Shaw et al. [39]	Clinical	Nurse	NA	Formal	NA	NA	Yes	Positive
Shaw et al. [39]	Clinical	Physician Assistant	NA	Formal	Achievement, innovative	Building coalitions	NA	NA
Shaw et al. [39]	Clinical	Physician	NA	Formal	NA	NA	NA	NA
Shaw et al. [39]	Hybrid (clinical/admin)	Physician	NA	Informal	Achievement, innovative, persistence, persuasive	Building coalitions	NA	Positive
Sloan et al. [40]	Hybrid (clinical/admin)	Physician	Varies	NA	Achievement, innovative	Rational arguments	Yes	Positive
Verhoeven et al. [27]	Clinical	Nurses	NA	NA	NA	Rational arguments	Yes	Positive
Wright et al. [54]	Admin	Multiple (various)	Yes	NA	NA	NA	Yes	NA
Yuan et al. [30]	NA	Multiple (various)	NA	NA	NA	Assertive actions, individualized consideration	Yes	Positive and negative
Yusof [64]	Clinical	Multiple (various)	NA	Yes	NA	Building coalitions	No	Positive
Zandieh et al. [62]	Clinical	Physician	NA	NA	NA	NA	Yes	Positive

### Champion characteristics

#### Personality characteristics

We identified 20 articles representing 19 studies that described characteristics of champions similar to those identified by Howell and Higgins [11], including achievement [31–40]; persuasiveness [36–39, 41–44]; persistence [10, 43, 45, 46]; innovativeness [31, 35, 36, 38–40, 43, 46–48]; charisma [36, 39, 47]; enthusiasm [35–37, 39, 40]; assertiveness [28, 36, 39, 47]; risk-tolerance [28, 38, 46]. Furthermore, many of the champions in our review demonstrated combinations of the personality characteristics mentioned above, such as achievement and innovativeness [28, 32, 33, 35, 39, 40]; persistence and persuasiveness [29, 41, 43, 44]; achievement, innovativeness, and persuasiveness [31, 38, 39]; and achievement, innovativeness, persistence, and persuasiveness [31, 36, 39].

#### Organizational role

Champions held formal leadership roles such as medical director, nurse manager, chief information officer, chief medical information officer, practice manager, or office manager [24, 28, 31–33, 39, 41, 43, 47, 49–53]. However, there were also cases where champions did not have a formal leadership role in the organization [25–28, 30–34, 39, 43, 44, 46–48, 50–52, 54–56]. Some champions held dual roles with administrative and clinical responsibilities [24, 32, 33, 38–41, 51], or clinical and technical responsibilities [36, 46]. Furthermore, in some studies champions were described as emergent [26, 30, 32, 33, 38, 39, 52], whereas in other studies champions were formally appointed [24, 28–30, 32, 36, 39, 42, 45, 50, 51, 55, 57, 58]. The study by Yuan and colleagues, which included both emergent and appointed champions, identified this difference as a key contextual factor influencing the champion’s approach [30].

#### Experience and training

Experience and/or training may be influenced by the organizational role of the champion (e.g., clinicians must have clinical training). Over half of the champions in our study were physicians; however, other studies identified pharmacists, physician’s assistants [24, 39, 46], and nursing staff [27, 30, 38, 39, 51, 52, 55, 59, 60]. Finally, some studies described champions without clinical training [29, 33, 39, 54]. Some champions had both clinical and technical experience or training, such as technologically savvy physicians [28, 29, 36, 42, 45, 46, 48, 51, 54, 55]. Several studies described champions with various levels of experience and training with information technology [40, 47, 59]; however, the majority of studies did not identify whether champions had specific experience or training with information technology [24, 27, 32, 33, 35, 37–39, 41, 44, 50, 52, 56, 57, 60–62].

#### Types of champions

Our findings related to personality characteristics, organizational role, and experience and training suggest different categories of HIT champions: clinical, technological, administrative, and hybrid. Clinical champions have clinical training, perform clinical activities within the organization, and interact with other clinical users of the innovation. Technological champions have IT-specific expertise and are able to maintain the technological infrastructure of the innovation and/or provide technical assistance to users of the innovation. Administrative champions are found within various levels of the organization—senior leadership (e.g., CIO); mid-level management (e.g., department manager); and front-line staff (e.g., nurse). These champions may perform such functions as strategy development, program administration, or project coordination. Hybrid champions are individuals who illustrate two or more of the aforementioned champion categories. These individuals typically hold dual roles within the organization and/or have multidisciplinary training or experience. This concept of a hybrid champion is similar to the “special people” in Ash and colleagues’ study [31]. Such individuals are able to communicate effectively with clinical, IT, and administrative personnel. For example, a hybrid champion may train peers to use the HIT effectively, work with IT staff to customize the HIT, and work with administrators to overcome barriers to the implementation (e.g., policy/bureaucratic barriers, lack of buy-in from senior leadership, front-line staff resistance). Recognizing that there are different types of champions is important for assessing champion characteristics, tactics, and impact because organizational members' expectations of champions may vary Andre et al. [63], as might the champions' goals, depending on the champion type.

### Champion influence tactics

Several studies report various influence tactics used by champions that are similar to those identified by Howell and Higgins [11]: building coalitions [28, 31–33, 36, 38, 39, 43, 47–49, 57, 59], appealing to higher authority [33, 38], bargaining [28, 29, 38], performing clandestine actions [43], presenting rational arguments [27, 28, 30, 33, 36, 38, 40, 42, 44, 47, 64], applying sanctions [38, 52], using friendliness and ingratiation [36, 38, 52], assertive actions [28, 36, 39], individualized consideration [24, 30, 36, 44]; and intellectually stimulating other organizational members [43]. Champions also spent extensive time engaging in workflow redesign [28, 46] and overcoming technical challenges [28, 30, 33, 36, 64]. Some champions used multiple combinations of influence tactics such as appealing to higher authority and rational arguments [31, 33, 47]; building coalitions, rational arguments, ingratiation and friendliness [36]; building coalitions and clandestine actions [43]; making rational arguments and bargaining [28]; and applying sanctions, ingratiation and friendliness [52]. In Paré and colleagues’ study of the implementation of a patient charting system in the burn center of a large, non-profit teaching hospital, a champion built coalitions, appealed to higher authority, applied sanctions, bargained, and performed assertive actions [38]. Another study suggests that CIO champions may employ various tactics, depending on the level of technology knowledge held by the hospital board [28]. Furthermore, when HIT implementation involves multiple organizations (e.g., a health information organization and a health care delivery system), having a champion that spans the organizational boundaries could be a driver of project success, as the champion brings an understanding of the vision and capacity of both organizations [49]. Finally, Yuan and colleagues’ study classifies some champion behaviors as supportive of implementation (e.g., reporting problems to someone in a position to fix it and employing teaching strategies that promoted “learning by doing”) and other behaviors as causing implementation challenges (e.g., losing patience with coworkers and creating workarounds that undermined appropriate use of the HIT) [30].

### Management support for the champion

Study results suggest that organizations with innovative cultures fostering collaboration and experimentation are supportive of champions during HIT implementation [36]. Specific actions of organizational leaders that illustrate support for champions include being closely involved and affiliated with the new HIT [39, 43, 44, 60, 61], setting clear expectations that organizational members use the new HIT [58], supporting pilot projects to allow experimentation with the technology [48], prominently mentioning the new HIT in strategy and policy plans [44], providing access to organizational IT support [60], and recognizing and rewarding champions [43]. Some studies reported insufficient support for the champions’ efforts, including perceived lack of system-level leadership support for implementation activities [37], insufficient organizational slack [37], or purchasing equipment but not financially investing in program activities [50].

Information technology-related knowledge of top leadership appears to play a key role in the support provided to champions. For example, Leidner et al. focused on the extent to which a governing board’s IT-knowledge influences CIO-level innovation champions in hospitals [28]. They found four types of CIO innovators: board-informing, board-constrained, board-invisible, and board-driven. Board-informing CIO innovators work to educate their board on the strategic implications of potential IT solutions. The support CIOs receive appears to be influenced by the extent to which those implications are valued. The board-constrained CIO innovator works with a board that is relatively IT-savvy and comfortable assessing IT opportunities for themselves. These CIOs generally follow the discretion of the board and receive little support for innovation. Board-invisible CIO innovators operate generally independently of the board, which is not particularly IT-savvy. These CIOs can be well supported for innovation if they develop the trust of their board. Finally, board-driven CIO innovators work with IT-savvy boards that are eager to innovate. CIOs in this environment are well supported in efforts to innovate; however, they may face challenges related to reigning in the board’s expectations for IT. In summary, organizational culture and support from leadership and top management appear to be key contextual conditions that affect the priorities, actions, and impact of champions on implementation.

### Champion impact

Our review revealed that studies have assessed three general types of HIT champion impacts: (1) impact on the implementation process of a specific HIT innovation [39, 43, 55]; (2) impact on usage behavior or overall success of a specific HIT innovation [26, 27, 32, 33, 36, 38, 39, 43, 44, 49, 51, 55, 59, 60, 64]; and (3) impact on general organizational-level innovativeness [28, 39]. The main thrust of the third type of impact is that a champion’s success or failure can have implications beyond the scope of the particular implementation for which they champion. In other words, a champion not only can positively or negatively affect the process and outcome of a particular HIT implementation, but they also can affect the organization’s overall experience and capacity related to innovation.

Both quantitative and qualitative approaches have been used to assess impact; however, we found qualitative approaches to be much more commonly used. Only a handful of studies used quantitative methods to assess relationships between the presence of a champion and impacts [26, 51, 60]. Regardless of the methodological approach, most studies reported that the presence of a champion was associated with a positive impact on the implementation process and success of various HIT projects, such as an electronic medical record [36], clinical decision support [47], and e-prescribing [42]. However, some findings indicate that a champion is not necessarily sufficient for successful implementation [27, 43]. Furthermore, some champions may have a greater impact than others. In an analysis of two implementation settings, one of which had a more successful implementation than the other, Yuan and colleagues found variation in champion impact with respect to supporting their peers (proactively versus reactively); depth of explanation (emphasizing why actions had to be performed versus demonstrating how to accomplish tasks but not explaining the logic behind these actions); framing (using positive frames to diffuse tension versus using neutral frames); and information-sharing (consistently sharing information about the HIT with all users versus limiting the spread of information to individuals they interacted with the most) [30].

Some studies suggest that multiple champions may be necessary for a positive impact on implementation. For example, in Paré’s detailed study of the implementation of a patient charting system, the medical director of the burn center played key roles in adoption and implementation. He exhibited many of the personality characteristics we examined, and used several tactics including building coalitions, appealing to higher authority, bargaining, applying sanctions, and assertive actions. However, nurse managers with characteristics such as innovativeness, persuasiveness, and persistence were also important champions in implementation through the presentation of rational arguments to clinical staff [38]. Furthermore, Shaw and colleagues’ multiple-case-study provides examples of eight primary care organizations with no champion; a project champion or an organizational change champion; or a project champion and an organizational change champion [39]. This multiple-case-study suggests that both project and organizational-change champions may be important for implementing and sustaining quality improvement efforts. Although individual project champions may be sufficient for leading a specific change effort, any gains made in the effort could be jeopardized if the effort does not align with the organization’s broader plan and future vision for change, thus necessitating a high-level administrative champion.

### Comparison of quantitative and qualitative study results

The quantitative studies in our review focused on the impact of the champion in relation to an organization’s readiness for IT implementation, an organization’s overall innovativeness in the area of IT, and the adoption of a specific technology by organizational members. In terms of organizational readiness, results from Paré et al. [61] were mixed regarding whether having a project champion increases readiness for an IT-driven change. The study evaluated two different settings and technologies: a nursing information system in an oncology and palliative care unit and an EHR implementation in a large teaching hospital. Having a project champion was not a significant contributor to organizational readiness for the nursing system but was significant for the EHR (path coefficient = 0.23, *p* < 0.05). Regarding adoption and implementation of specific technologies, results from Hao et al. [26] show that an individual in a group with an opinion leader (i.e., an individual identified by the health system as an early adopter of the technology) is more likely to adopt hand-held device in practice than a user who has no opinion leader in his or her group (odds ratio = 3.125, *p* < 0.05). Similarly, Sharkey et al. [60] found that the presence of a champion was correlated with higher level of implementation of tools for clinical decision support in nursing homes, that is, having a collaborative approach, being highly engaged in ways that CDS report data are used, and implementing three or more process improvements facility-wide (Spearman correlation coefficient = 0.75, *p* < 0.01). Regarding impact on overall innovativeness related to IT, results from Leidner et al. [28] suggest that CIO champions demonstrating strategic leadership—that is, whether other executives perceive the CIO to be visionary and able to weave business and IT strategies together—influences whether the hospital is perceived as a leader in HIT.

In general, both the quantitative and qualitative studies in our review yielded somewhat mixed results regarding the impact of champions on HIT implementation; however, positive impacts are more commonly reported in both types of studies. Notably, as compared to the quantitative results, qualitative studies in our review provided more detail about the characteristics, tactics, and management support of the champions being studied and, therefore, are better able to elucidate how a champion contributed to a particular implementation outcome, for example, through face-to-face communication about the value of the technology [24, 34]. These studies provide a basis for developing hypotheses to test in larger studies analyzing determinants of successful HIT implementation using quantitative measures.

## Discussion

The number of empirical studies focusing on HIT champions is relatively modest, and existing studies employ a variety of methodological and measurement approaches. Therefore, identifying evidenced-based practices for HIT champions remains a goal for future research, particularly in light of the complex role of champions in implementation. Nevertheless, our review revealed some important insights.

In general, champions appear to contribute to the successful implementation of many HIT projects. However the extent to which HIT projects fail even with a champion and why such failures occur is less clear. Also unclear is whether all organizations require a champion for successful HIT project implementation. More specifically, we currently do not know enough about the conditions under which (1) a health IT champion is needed, (2) multiple champions are needed, and (3) an appointed champion—as opposed to an emergent champion—can be successful. A number of qualitative studies in our review provide rich details suggesting that organizational climate, resources, technical capability, workflow modifications, and resistance to change influence the characteristics, tactics, management support, and impacts of champions. However, many of our studies provided little detail about characteristics of the organizations or the organizational members undergoing implementation, let alone the circumstances under which the implementation was occurring. Therefore, the extent to which the organizational context may influence the need for a champion (including the number and type of champions); the phase of the IT project that requires a champion (e.g., adoption-decision, pre-implementation planning, etc.); the tactics a champion should employ; and how management can best support the champion(s) remain largely unknown. For example, an organization already undertaking multiple implementation efforts may require a top-level administrator to communicate the strategic value of a new HIT change, including how the effort aligns with broader organizational priorities and change efforts. The organization may also need a team of champions representing various roles to serve as subject matter experts and to assist with training peers to use the system effectively. Multiple-case-study approaches, similar to that used in Shaw et. al., could be useful for assessing relationships between organizational context and champion characteristics, tactics, and impacts [39].

Robust studies that focus on champions and HIT implementation effectiveness, require appropriate theories to guide hypothesis generation. The model we employed to guide our review was useful for characterizing the nature of the literature; however, we believe it would require modification in order to inform future research on HIT champions. In general, more attention is needed to developing theory that predicts champion impacts given the number and characteristics of champions, tactics they employ, characteristics of the HIT being implemented, and organizational context within which the implementation is occurring. Theory development should account for different types and levels of impact (e.g., impacts on user adoption, user productivity, and organizational capacity for future implementations). Without theory-informed studies, our understanding of the conditions under which champions promote successful implementation will be dependent upon the results of a small number of studies that may not account for interactions between some of the most influential organizational, individual, and technological factors.

Although our study focused on HIT, our findings complement studies from the information systems literature that are not specific to health care. Specifically, information systems research has demonstrated that change agents have varying characteristics and roles during implementation [65]; employ various influence tactics [66, 67]; frequently depend on management support, alliances, organizational context [68, 69]; and are associated with positive and negative implementation outcomes [65]. Also, characteristics, roles, influence tactics, support, organizational context, and outcomes have been found to be associated with the complexity of information system implementation [70, 71]. Finally, studies have suggested that complex adaptive system theory holds promise for future work related to developing HIT implementation frameworks [72, 73].

### Limitations

Our review has a few limitations. First, we identified a relatively small number of studies focused on HIT champions. Second, many of these studies did not focus explicitly on HIT champions but rather studied HIT implementation in general and included champions as a part of that analysis or as an emergent finding. Third, studies included in our review varied in terms of their level of detail about champions and their methods (with most studies being qualitative). Given the various ways in which champions have been defined and studied, abstracting data for each study required substantial discussion between the authors and an iterative process. However, we believe having a team comprised of researchers with qualitative data analysis experience enhanced the validity of our findings. Finally, we believe these limitations are not uncommon for a scoping review designed to describe the nature of the literature on a broad research question.

## Conclusions

Our review was carried out to assess the extent and scope of research on HIT champions for the purpose of informing future research. We identified 42 studies pertinent to HIT champions. Our findings suggest that additional research is needed to analyze the characteristics, influence tactics, and management support needed for different categories of champions. Future work should view champions as one component within the organizational infrastructure for HIT implementation. Answers to questions about who champions should be, when they should be engaged, what they should do, how management can support their efforts, and what their impact will be are likely dependent upon other aspects within the organization. Because many organizations currently appoint champions for HIT projects, rather than allowing them to emerge, researchers should focus on developing evidence-based frameworks and/or tools that assist with identifying which aspects of an implementation require such a champion’s efforts, the experience and skills the champion needs in order to perform effectively, and the types of management support that the champion will need.
